# Development and validation of a nomogram for early prediction of macrolide-unresponsive Mycoplasma pneumoniae pneumonia in children

**DOI:** 10.3389/fped.2025.1695974

**Published:** 2025-11-20

**Authors:** Hui Liu, Rina Sa, Yang Xin, Meilan Zhang, Hai Du

**Affiliations:** 1 Graduate School, Baotou Medical College, Baotou, China; 2 Department of Radiology, Ordos Central Hospital, Ordos, China; 3 Department of Pediatrics, Ordos Central Hospital, Ordos, China; 4 Ordos Clinical Medical College, Inner Mongolia Medical University, Ordos, China

**Keywords:** Mycoplasma pneumoniae pneumonia, macrolide-resistant, children, nomogram, prediction model, tree-in-bud sign, neutrophil, platelet-to-lymphocyte ratio

## Abstract

**Background:**

Mycoplasma pneumoniae (MP) is a leading cause of community-acquired pneumonia in children, with a significant increase in incidence following the COVID-19 pandemic. The emergence of macrolide-resistant M. pneumoniae (MRMP) has complicated treatment, leading to the concept of macrolide-unresponsive M. pneumoniae pneumonia (MUMPP), defined as lack of improvement after 72 h of macrolide therapy. Early identification of MUMPP is critical for timely intervention and improved outcomes. This study aimed to develop and validate a nomogram for early prediction of MUMPP in children.

**Methods:**

We conducted a retrospective study involving 278 pediatric patients with MP pneumonia, divided into training (*n* = 188) and validation (*n* = 90) sets. Demographic, clinical, laboratory, and chest CT imaging data were collected. Univariate and multivariate logistic regression analyses were used to identify independent predictors of MUMPP. A nomogram was constructed and validated using receiver operating characteristic (ROC) curves, calibration plots, and decision curve analysis (DCA).

**Results:**

Six independent predictors were identified: tree-in-bud pattern, neutrophil-value, lymphocyte-value, creatine kinase (CK), platelet-to-lymphocyte ratio (PLR), and male gender. The nomogram demonstrated strong discriminatory power, with area under the curve (AUC) values of 0.838 (95% CI: 0.779–0.897) in the training set and 0.835 (95% CI: 0.752–0.918) in the validation set. Calibration and DCA confirmed good clinical utility.

**Conclusion:**

We developed and validated a simple-to-use nomogram for predicting MUMPP in early stage. The nomogram demonstrates strong discriminatory power and calibration, and may be a practical tool for clinical practice.

## Introduction

Mycoplasma pneumoniae pneumonia (MPP) accounts for approximately 10%–40% of community-acquired pneumonia (CAP) in school-aged children (1). According to reports from the World Health Organization and multiple countries, the burden of respiratory diseases in children has increased significantly following the COVID-19 pandemic, with a notable rise in the incidence of MPP (2). Macrolides are the primary antibiotics used to treat MPP. However, compared to the pre-pandemic period, macrolide-resistant *Mycoplasma pneumoniae* (MRMP) has become increasingly prevalent worldwide, particularly in Asian countries. Resistance rates have reached approximately 69.67% in South Korea and as high as 90% in Japan and China (3–5). This trend poses a serious threat to children's health, often leading to prolonged fever, extended hospital stays, increased severity of cases, and challenges in antibiotic selection (6). In response, the 2023 Chinese Guidelines for the Diagnosis and Treatment of MPP in Children introduced the concept of “Macrolide-Unresponsive *Mycoplasma pneumoniae* Pneumonia (MUMPP), defined as cases showing no clinical or radiological improvement after 72 h of macrolide treatment” (7). MUMPP can serve as an early indicator of MRMP. Therefore, early identification of MUMPP and timely adjustment of antibiotics are crucial for shortening the disease course and reducing the risk of severe complications and sequelae (8).

Imaging plays an increasingly important role in the diagnosis and evaluation of pneumonia. Although chest x-ray remains the first-line imaging method for assessing CAP, its findings in MPP are often non-specific and may be missed due to anatomical overlap. In contrast, computed tomography (CT) provides clearer visualization of parenchymal and interstitial lung abnormalities—such as bronchial wall thickening and bronchiectasis—which correlate well with pathological changes (9, 10). With higher spatial and density resolution, CT offers a more reliable basis for accurately assessing the extent and pattern of lung involvement in children, thereby guiding clinical decision-making.

The nomogram is an intuitive and user-friendly tool for multivariate prediction modeling. It integrates key predictive factors into a visual risk assessment model, facilitating individualized risk evaluation and supporting clinical decision-making (11). Existing prediction models for refractory MPP (RMPP), severe MPP (SMPP), and necrotizing MPP (NMPP) have identified several independent risk factors, including peak body temperature, pleural effusion, extrapulmonary complications, neutrophil ratio, C-reactive protein (CRP), erythrocyte sedimentation rate (ESR), procalcitonin (PCT), D-dimer, lactate dehydrogenase (LDH), and albumin (ALB) (12–14). However, few studies have focused on developing clinical prediction models specifically for MUMPP. Current diagnosis relies heavily on symptomatic presentation and isolated laboratory findings, which vary based on physicians' experience and subjective judgment, leading to inconsistent and potentially biased outcomes. Hence, there is an urgent need to develop an effective, economical, and rapid diagnostic tool to support timely and accurate treatment.

This study aimed to identify factors for the early prediction of MUMPP and to develop a simple-to-use nomogram.

## Materials and methods

### Study subjects and design

This retrospective research was conducted in adherence to the Declaration of Helsinki. Ethical approval, which included a waiver for individual consent, was obtained from the Ethics Committee of Ordos Central Hospital (No. 2025-406).

**Figure 1:**
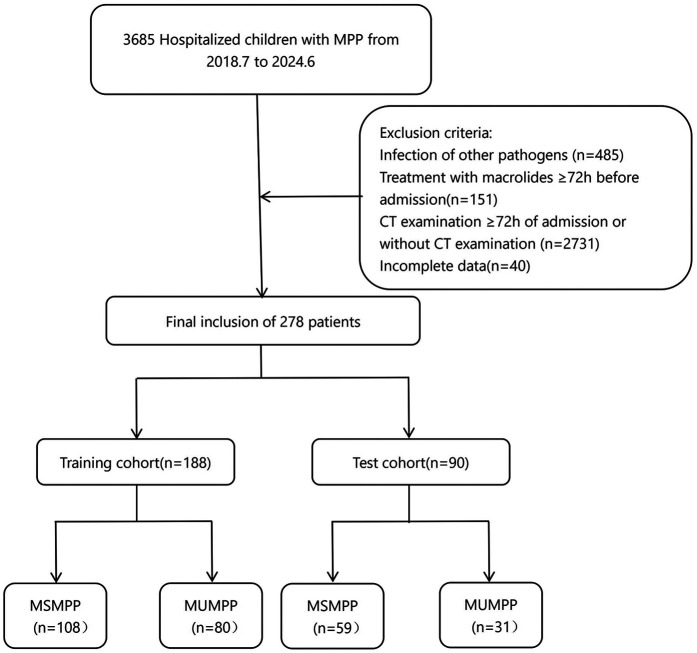
Patient screening workflow for this study. MUMPP, macrolide-unresponsive M. pneumonias pneumonia, MSMPP macrolide-sensitive M. pneumonias pneumonia.

In this study, 278 children diagnosed with MPP and hospitalized for treatment were enrolled. Among them, 188 cases from the main campus of Ordos Central Hospital between July 2018 and June 2024 were assigned to the training set. An additional 90 cases from the branch campus between April 2019 and June 2024 were collected as the test set, (Figure 1).

Inclusion criteria: (1) were under the age of 14 years; (2) clinical signs and symptoms suggestive of CAP, including fever, cough, adventitious breath sounds (rales or diminished breath sounds), and new infiltrates on chest x-ray or CT; (3) positive serological testing for MP-IgM (serum anti-MP IgM titer ≥1:160 or a ≥ 4-fold increase in antibody levels), or positive MP-RNA or MP-DNA detection in throat swab or bronchoalveolar lavage fluid (BALF); (4) noncontrast chest CT performed within 72 h of admission.

The following tests were used to rule out other respiratory infections and tuberculosis: purified protein derivative (PPD) test, blood culture, pleural fluid culture, nasopharyngeal aspirate/swab culture, viral antigen testing (for respiratory syncytial virus, influenza virus, adenovirus, and parainfluenza virus), and serology for Chlamydia pneumoniae and Legionella pneumophila.

Exclusion criteria included: (1) macrolide antibiotic use for ≥72 h prior to admission; (2) foreign body aspiration; (3) chronic respiratory diseases; (4) malignancy, solid organ transplantation or surgery, immunodeficiency, or use of immunosuppressive drugs; (5) congenital or inherited metabolic disorders and other underlying conditions; (6) incomplete medical records; (7) poor patient cooperation leading to significant imaging artifacts. Furthermore, patients with any missing values in the studied variables were excluded from the final analysis to ensure a complete dataset for model development.

Depending on whether patients had persistent fever, unresolved clinical symptoms, and worsening or stagnant pulmonary imaging following 72 h of macrolide therapy, they were classified into Macrolide-Sensitive Mycoplasma pneumoniae Pneumonia (MSMPP) group and unresponsive (MUMPP) group.

### Data collection

We collected data from the electronic health medical record system, including general demographics, clinical features, laboratory results at admission.

1. Demographic data included gender, age, and ethnicity.
2. Clinical features encompassed presence of fever, cough, wheezing, pre-admission cough duration (PCD), pre-admission fever duration (PFD), the highest body temperature (T), heart rate (HR) and respiratory rate (R) on admission and length of hospital stay.
3. Laboratory results consisted of white blood cell count (WBC), neutrophil count (NEUT_value), neutrophil percentage (NEUT), lymphocyte count (LYMPH_value), lymphocyte percentage (LYMPH), monocyte count (MONO), platelet count (PLT), C-reactive protein (CRP), alanine aminotransferase (ALT), aspartate aminotransferase (AST), lactate dehydrogenase (LDH), creatine kinase (CK), creatine kinase isoenzyme (CK-MB), and albumin (ALB). Additionally, ratios of certain serum markers were evaluated, including ratio of neutrophil-value to lymphocyte-value (NLR), ratio of platelet to lymphocyte-value (PLR), ratio of lymphocyte-value to CRP (LCR), and LDH-to-albumin ratio (LAR).

All patients underwent noncontrast chest CT within 72 h of admission.

### CT examinations

CT scans were performed while the children were in a calm state. For uncooperative patients, appropriate sedation was administered to ensure successful completion of the examination. Scans were conducted using GE Lightspeed VCT 64-slice, GE Discovery CT 750HD, GE Revolution CT 256-slice, and SIEMENS SOMATOM Force dual-source CT scanners. Scanning parameters were as follows: tube voltage 80–120 kV, tube current 50–350 mA, slice thickness 5 mm, slice interval 5 mm, and reconstructed image thickness 1.25 mm. The CT scan was performed from the lung apex to the lung base. For mediastinal window settings, the window width was 350 HU and window level 40 HU; for lung windows, the window width was 1,500 HU and window level −600 HU.

### Imaging analyses

CT images were initially evaluated by a radiologist with 10 years of experience in thoracic imaging and reviewed by another with more than 15 years of experience. In case of disagreement, a consensus was reached through discussion or consultation with a senior attending radiologist. Recorded CT features included lesion location, number of involved lobes, unilateral or bilateral involvement, bronchial wall thickening, tree-in-bud sign, air bronchogram, interlobular septal thickening, consolidation pattern (patchy, segmental, lobar), consolidation with ground-glass opacity (GGO), mosaic pattern, with or without pleural effusion.

### Statistical analysis

All statistical analyses in this study were performed using R software (version 4.5.1). Continuous variables following a normal distribution were presented as mean ± standard deviation and compared using the Student's *t*-test. Non-normally distributed continuous variables were summarized as median with interquartile range [M (P25, P75)] and compared using the Mann–Whitney *U* test. Categorical variables were expressed as frequency (percentage) and compared using the chi-square test.

For variable selection, potential predictors were first screened by univariate logistic regression, retaining those with a significance level of *P* < 0.05. Multicollinearity among these selected variables was then assessed using the variance inflation factor (VIF), and variables with VIF > 5 were iteratively removed. The remaining variables with low collinearity were entered into a multivariate logistic regression model. A stepwise backward elimination method was applied, with inclusion and exclusion criteria set at *P* < 0.05 and *P* > 0.10, respectively, to identify the final independent predictors and construct the prediction model. In addition to VIF assessment, correlation analysis was conducted to further verify the absence of severe multicollinearity among the final predictors in the model. Furthermore, for continuous variables included in the final model, four-knot restricted cubic spline analysis was employed to assess the presence of nonlinear relationships with the logit-transformed outcome variable.

The predictive performance of the model was evaluated using the area under the receiver operating characteristic (ROC) curve, accuracy, sensitivity, and specificity. Predictive consistency was assessed using calibration curves, and clinical utility was determined via decision curve analysis. All statistical tests were two-sided, *P* < 0.05 was considered statistically significant.

## Results

### Clinical characteristics

Among the 278 pediatric cases, there were 111 cases of MUMPP and 167 cases of MSMPP. Including 125 boys (44.96%) and 153 girls (55.04%). The median age was 7.23 ± 2.64 years, and the average length of hospital stay was 8.20 ± 3.10 days. Mycoplasma pneumoniae pneumonia cases were detected throughout the year, with a peak incidence observed in the fourth quarter. Table 1 summarizes the characteristics of children in the training and test sets at admission. No significant differences were observed in gender or ethnicity (*P* > 0.05). Clinical manifestations, routine blood tests, and biochemical markers within 24 h of admission were compared between the two groups. In the training set, compared with the MSMPP group, the MUMPP group had a longer duration of fever before admission, higher levels of NEUT, NLR, CRP, AST, CK, LDH, PLR, and LAR, as well as lower respiratory rate, LYMPH_value, LYMPH, MONO, ALB, and LCR. In the test set, the MUMPP group exhibited a longer duration of cough before admission, and significant differences were found in NEUT, LYMPH, LYMPH_value, NLR, CK, and PLR (*P* < 0.05). Notably, in both the training and test sets, children in the MUMPP group were older and had a higher incidence in the fourth quarter. The other results showed no significant differences (*P* > 0.05).

**Table 1:** Baseline characteristics of patients in the training and test cohort.

Feature	Training cohort			*p*	Test cohort			*p*
	ALL	MUMPP	MSMPP		ALL	MUMPP	MSMPP	
Age (years)	7.11 ± 2.61	7.79 ± 2.23	6.60 ± 2.76	0.003	7.50 ± 2.69	8.45 ± 1.80	7.00 ± 2.95	0.022
hospital_days	8.74 ± 3.42	8.81 ± 3.37	8.69 ± 3.47	0.815	7.07 ± 1.87	7.00 ± 2.32	7.10 ± 1.59	0.482
PCD (days)	6.09 ± 4.71	6.16 ± 3.31	6.03 ± 5.53	0.181	5.91 ± 2.35	6.45 ± 1.96	5.63 ± 2.50	0.045
PFD (days)	5.34 ± 3.33	5.79 ± 2.48	5.00 ± 3.82	0.006	5.51 ± 2.25	5.65 ± 2.26	5.44 ± 2.27	0.664
T (°C)	37.18 ± 1.00	37.20 ± 1.01	37.17 ± 1.00	0.968	37.47 ± 0.88	37.63 ± 1.04	37.39 ± 0.78	0.462
HR	104.28 ± 12.81	102.06 ± 10.28	105.93 ± 14.22	0.091	113.24 ± 12.99	111.19 ± 14.62	114.32 ± 12.04	0.280
R	22.28 ± 4.19	21.91 ± 4.19	22.55 ± 4.19	0.011	24.17 ± 3.23	23.71 ± 2.97	24.41 ± 3.36	0.222
WBC (×10^9^/L)	7.39 ± 2.95	7.35 ± 3.35	7.42 ± 2.64	0.467	6.81 ± 2.15	6.70 ± 2.27	6.87 ± 2.10	0.802
NEUT (%)	64.12 ± 12.55	68.81 ± 10.98	60.64 ± 12.56	<0.001	65.77 ± 10.65	69.37 ± 9.52	63.87 ± 10.80	0.015
NEUT_value (×10^9^/L)	4.82 ± 2.37	5.30 ± 2.98	4.47 ± 1.71	0.117	4.58 ± 1.85	4.54 ± 1.91	4.60 ± 1.83	0.905
LYMPH (%)	26.70 ± 10.69	22.67 ± 9.51	29.68 ± 10.57	<0.001	25.29 ± 9.66	21.66 ± 7.72	27.19 ± 10.09	0.011
LYMPH_value (×10^9^/L)	1.93 ± 1.22	1.51 ± 0.69	2.25 ± 1.42	<0.001	1.69 ± 0.85	1.35 ± 0.53	1.87 ± 0.94	0.006
MONO (×10^9^/L)	0.56 ± 0.79	0.47 ± 0.25	0.63 ± 1.02	0.022	0.47 ± 0.17	0.46 ± 0.20	0.47 ± 0.15	0.868
NLR	3.16 ± 2.21	4.14 ± 2.77	2.43 ± 1.28	<0.001	3.15 ± 1.57	3.66 ± 1.65	2.88 ± 1.47	0.034
PLT (×10^9^/L)	275.10 ± 87.67	262.10 ± 91.84	284.72 ± 83.57	0.097	272.52 ± 81.06	267.97 ± 88.96	274.92 ± 77.29	0.658
CRP (mg/L)	23.63 ± 29.01	27.79 ± 30.83	20.54 ± 27.33	0.0119	25.23 ± 28.43	21.15 ± 16.93	27.38 ± 32.84	0.405
ALT (U/L)	23.47 ± 33.20	24.26 ± 26.60	22.89 ± 37.45	0.146	15.99 ± 11.41	15.74 ± 9.93	16.12 ± 12.19	0.627
AST (U/L)	36.90 ± 18.19	40.19 ± 21.57	34.47 ± 14.85	0.007	29.59 ± 11.16	27.84 ± 7.86	30.51 ± 12.52	0.334
CK (U/L)	130.29 ± 174.70	183.55 ± 251.53	90.83 ± 54.19	<0.001	142.57 ± 147.67	163.26 ± 132.39	131.69 ± 155.07	0.033
CK_MB (U/L)	25.61 ± 12.50	27.24 ± 14.07	24.41 ± 11.12	0.165	19.48 ± 6.57	17.38 ± 4.28	20.59 ± 7.29	0.076
LDH (U/L)	332.64 ± 111.63	376.63 ± 130.82	300.05 ± 81.38	<0.001	308.79 ± 82.55	300.55 ± 63.20	313.12 ± 91.29	0.935
ALB (g/L)	42.40 ± 3.20	41.47 ± 3.42	43.09 ± 2.85	<0.001	40.61 ± 2.93	40.49 ± 3.15	40.67 ± 2.83	0.786
PLR	176.63 ± 94.65	203.14 ± 104.82	156.99 ± 81.42	<0.001	188.55 ± 86.85	219.35 ± 96.20	172.36 ± 77.55	0.012
LCR	2.85 ± 30.72	0.22 ± 0.83	4.81 ± 40.49	<0.001	0.25 ± 0.95	0.12 ± 0.11	0.31 ± 1.17	0.664
LAR	7.98 ± 3.11	9.28 ± 3.74	7.01 ± 2.09	<0.001	7.71 ± 2.45	7.53 ± 2.03	7.81 ± 2.66	0.969
Sex				0.066				0.645
Female	105 (55.85)	38 (47.50)	67 (62.04)		48 (53.33)	15 (48.39)	33 (55.93)	
Male	83 (44.15)	42 (52.50)	41 (37.96)		42 (46.67)	16 (51.61)	26 (44.07)	
Ethnicity				0.573				1.0
Han	175 (93.09)	73 (91.25)	102 (94.44)		79 (87.78)	27 (87.10)	52 (88.14)	
Other	13 (6.91)	7 (8.75)	6 (5.56)		11 (12.22)	4 (12.90)	7 (11.86)	
Cough				0.613				1.0
No	2 (1.06)	null	2 (1.85)		1 (1.11)	null	1 (1.69)	
Yes	186 (98.94)	80 (100.00)	106 (98.15)		89 (98.89)	31 (100.00)	58 (98.31)	
Wheezing				0.613				1.0
No	186 (98.94)	80 (100.00)	106 (98.15)		89 (98.89)	31 (100.00)	58 (98.31)	
Yes	2 (1.06)	null	2 (1.85)		1 (1.11)	null	1 (1.69)	
Fever				0.358				1.0
No	9 (4.79)	2 (2.50)	7 (6.48)		1 (1.11)	null	1 (1.69)	
Yes	179 (95.21)	78 (97.50)	101 (93.52)		89 (98.89)	31 (100.00)	58 (98.31)	
Time_of_onset				0.005				0.002
1	51 (27.13)	21 (26.25)	30 (27.78)		21 (23.33)	3 (9.68)	18 (30.51)	
2	50 (26.60)	27 (33.75)	23 (21.30)		37 (41.11)	21 (67.74)	16 (27.12)	
3	25 (13.30)	3 (3.75)	22 (20.37)		7 (7.78)	2 (6.45)	5 (8.47)	
4	62 (32.98)	29 (36.25)	33 (30.56)		25 (27.78)	5 (16.13)	20 (33.90)	

MUMPP, macrolide-unresponsive M. pneumoniae pneumonia; MSMPP, macrolide-sensitive M. pneumoniae pneumonia; PCD, pre-admission cough duration; PFD, pre-admission fever duration; T, the highest body temperature; HR, heart rate; R, respiratory rate; WBC, white blood cell count; NEUT_value, neutrophil count; NEUT, neutrophil percentage; LYMPH_value, lymphocyte count; LYMPH, lymphocyte percentage; MONO, monocyte count; PLT, platelet count; CRP, C-reactive protein; ALT, alanine aminotransferase; AST, aspartate aminotransferase; LDH, lactate dehydrogenase; CK, creatine kinase; CK_MB, creatine kinase isoenzyme; ALB, albumin; NLR, neutrophil-to-lymphocyte ratio; PLR, platelet-to-lymphocyte ratio; LCR, lymphocyte-to-CRP ratio; LAR, LDH-to-albumin ratio.

### Chest CT imaging features

Figure 6A demonstrates the characteristic tree-in-bud sign on a chest CT image from a child with MUMPP. Chest CT imaging features are summarized in Table 2. Compared with the MSMPP group, the MUMPP group showed a significantly higher prevalence of tree-in-bud signs (*P* < 0.01). In the training set, mosaic pattern and lobar consolidation were also more common in the MUMPP group (*P* < 0.05), though these differences were not statistically significant in the test set. Other imaging features, including bronchial wall thickening, air bronchogram, interlobular septal thickening, segmental consolidation, patchy consolidation, bilateral lobar infiltration, and pleural effusion, did not differ significantly between the groups (*P* > 0.05).

**Table 2:** Radiological features of patients in the training and test cohort.

Feature	Training cohort			*p*	Test cohort			*p*
	ALL	MUMPP	MSMPP		ALL	MUMPP	MSMPP	
bronchial_wall_thickening (*n*, %)				0.322				0.026
No	92 (48.94)	43 (53.75)	49 (45.37)		36 (40.00)	7 (22.58)	29 (49.15)	
Yes	96 (51.06)	37 (46.25)	59 (54.63)		54 (60.00)	24 (77.42)	30 (50.85)	
air_bronchogram (*n*, %)				0.149				1.0
No	168 (89.36)	75 (93.75)	93 (86.11)		83 (92.22)	29 (93.55)	54 (91.53)	
Yes	20 (10.64)	5 (6.25)	15 (13.89)		7 (7.78)	2 (6.45)	5 (8.47)	
interlobular_septal_thickening (*n*, %)				0.102				1.0
No	182 (96.81)	75 (93.75)	107 (99.07)		84 (93.33)	29 (93.55)	55 (93.22)	
Yes	6 (3.19)	5 (6.25)	1 (0.93)		6 (6.67)	2 (6.45)	4 (6.78)	
tree_in_bud_sign (*n*, %)				0.001				<0.001
No	151 (80.32)	55 (68.75)	96 (88.89)		57 (63.33)	7 (22.58)	50 (84.75)	
Yes	37 (19.68)	25 (31.25)	12 (11.11)		33 (36.67)	24 (77.42)	9 (15.25)	
Consolidation_mixed_GGO (*n*, %)				0.397				0.941
No	161 (85.64)	66 (82.50)	95 (87.96)		83 (92.22)	28 (90.32)	55 (93.22)	
Yes	27 (14.36)	14 (17.50)	13 (12.04)		7 (7.78)	3 (9.68)	4 (6.78)	
mosaic pattern (*n*, %)				0.019				0.570
No	175 (93.09)	79 (98.75)	96 (88.89)		71 (78.89)	26 (83.87)	45 (76.27)	
Yes	13 (6.91)	1 (1.25)	12 (11.11)		19 (21.11)	5 (16.13)	14 (23.73)	
consolidation (*n*, %)				0.758				0.566
No	36 (19.15)	14 (17.50)	22 (20.37)		16 (17.78)	7 (22.58)	9 (15.25)	
Yes	152 (80.85)	66 (82.50)	86 (79.63)		74 (82.22)	24 (77.42)	50 (84.75)	
Patchy_consolidation (*n*, %)				1.0				1.0
No	178 (94.68)	76 (95.00)	102 (94.44)		79 (87.78)	27 (87.10)	52 (88.14)	
Yes	10 (5.32)	4 (5.00)	6 (5.56)		11 (12.22)	4 (12.90)	7 (11.86)	
segmental_consolidation (*n*, %)				0.055				1.0
No	87 (46.28)	44 (55.00)	43 (39.81)		43 (47.78)	15 (48.39)	28 (47.46)	
Yes	101 (53.72)	36 (45.00)	65 (60.19)		47 (52.22)	16 (51.61)	31 (52.54)	
lobar_consolidation (*n*, %)				0.002				1.0
No	150 (79.79)	55 (68.75)	95 (87.96)		77 (85.56)	27 (87.10)	50 (84.75)	
Yes	38 (20.21)	25 (31.25)	13 (12.04)		13 (14.44)	4 (12.90)	9 (15.25)	
Pleural_effusion (*n*, %)				0.111				1.0
No	110 (58.51)	41 (51.25)	69 (63.89)		71 (78.89)	24 (77.42)	47 (79.66)	
Yes	78 (41.49)	39 (48.75)	39 (36.11)		19 (21.11)	7 (22.58)	12 (20.34)	
Bilateral_lobar_infiltrates (*n*, %)				0.704				1.0
No	111 (59.04)	49 (61.25)	62 (57.41)		41 (45.56)	14 (45.16)	27 (45.76)	
Yes	77 (40.96)	31 (38.75)	46 (42.59)		49 (54.44)	17 (54.84)	32 (54.24)	

MUMPP, macrolide-unresponsive M. pneumoniae pneumonia; MSMPP, macrolide-sensitive M. pneumoniae pneumonia; GGO, ground-glass opacity.

### Variable selection

Univariate logistic regression analysis was performed on all features. Given that multiple indicators were associated with inflammatory response, 19 independent variables were first assessed for multicollinearity. Following the removal of highly correlated variables (LAR, LYMPH, and NLR), 16 low-collinearity features were retained for multivariate logistic regression. The analysis identified six independent predictors of MUMPP: sex, NEUT_value, LYMPH_value, CK, PLR, and tree-in-bud sign (*P* < 0.05) (Table 3, which only lists variables with statistical significance in the multivariate analysis).

**Table 3:** Results of multivariable logistic regression analysis.

Feature	Coef	OR	95% CI	*p*
Sex	−1.105	0.331	0.136–0.808	0.015
NEUT_value	0.412	1.510	1.044–2.185	0.029
LYMPH_value	−1.444	0.236	0.079–0.697	0.009
CK	0.007	1.007	1.001–1.013	0.014
PLR	−0.008	0.992	0.985–0.999	0.026
tree_in_bud_sign	1.810	6.111	1.876–19.914	0.003

Coef, coefficient; OR, odds ratio; CI, confidence interval; NEUT_value, neutrophil count; LYMPH_value, lymphocyte count; CK, creatine kinase; PLR, platelet-to-lymphocyte ratio.

**Figure 2:**
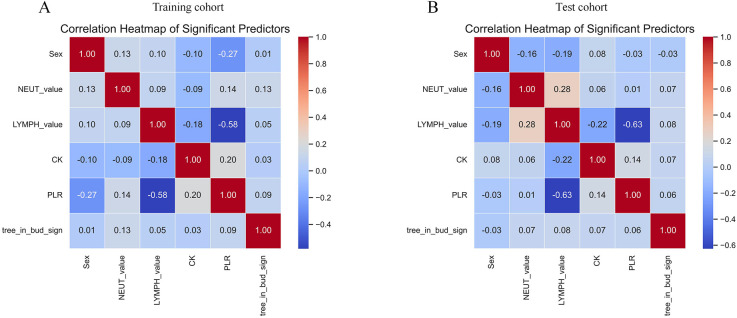
Heat map analysis of correlations between 6 variables. **(A)** Training cohort. **(B)** Test cohort. NEUT-value, neutrophil count; LYMPH-value, lymphocyte count; CK, creatine kinase; PLR, platelet-to-lymphocyte ratio.

A comprehensive correlation analysis confirmed the absence of substantial multicollinearity among the six final predictors (Figure 2). Evaluation of the continuous predictors using a four-knot restricted cubic spline (RCS) analysis revealed no significant nonlinear relationships with the logit of the outcome (*P* > 0.05) (Supplementary Tables S1, S2). Consequently, these variables were retained in their original linear form in the final model.

### Development and evaluation of the predictive nomogram

**Figure 3:**
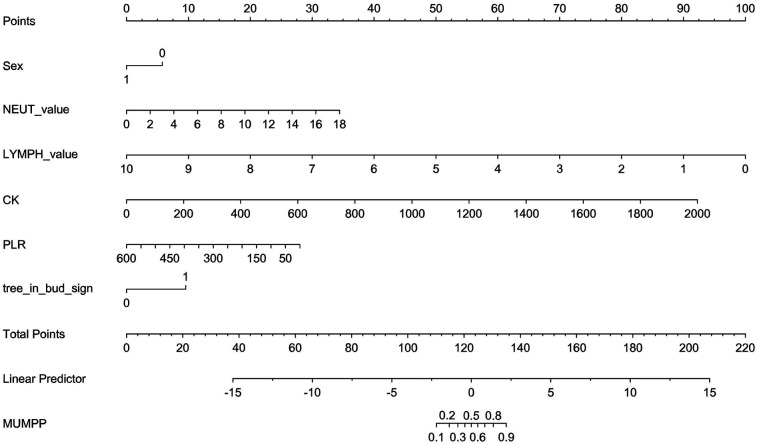
The nomogram to predict MUMPP. *Sex: 1 means female; 0 means male; tree_in_bud_sign: lmeans yes; 0 means no.

**Figure 4:**
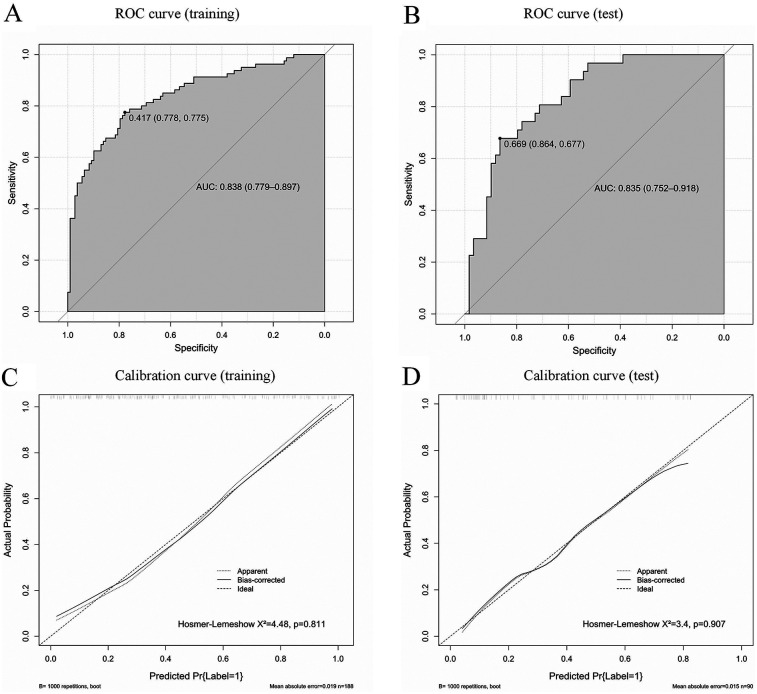
Receiver operating characteristic curves (ROC) of the model in the training set **(A)** and test set **(B)**; AUC, area under the curve. Calibration curves of the model in the training set **(C)** and test set **(D)**.

A nomogram was constructed for the early identification of MUMPP in children containing six independent predictors (Figure 3). A higher total score on the nomogram indicates a greater risk of MUMPP. In the training set, the nomogram showed an area under the curve (AUC) of 0.838 (95% CI: 0.779–0.897) (Figure 4A), while in the test set, the AUC was 0.835 (95% CI: 0.752–0.918) (Figure 4B), demonstrating robust discriminatory ability. Furthermore, the model exhibited robust specificity and PPV in the training cohort, a marked gain in sensitivity was achieved in the test cohort while maintaining strong specificity (Table 4). To rigorously evaluate the internal validity of the model, bootstrap validation with 1,000 resamples was performed, and the corresponding performance metrics are detailed in Supplementary Table S3.

**Table 4:** Diagnostic performance of model for MUMPP in training and test cohort.

Cohort	AUC (95% CI)	Accuracy	Sensitivity	Specificity	PPV	NPV	F1-score
Training cohort	0.838 (0.779–0.897)	0.766	0.538	0.935	0.860	0.732	0.662
Test cohort	0.835 (0.752–0.918)	0.756	0.742	0.763	0.622	0.849	0.676

AUC, area under the curve; CI, confidence interval; PPV, positive predictive value; NPV, negative predictive value.

**Figure 5:**
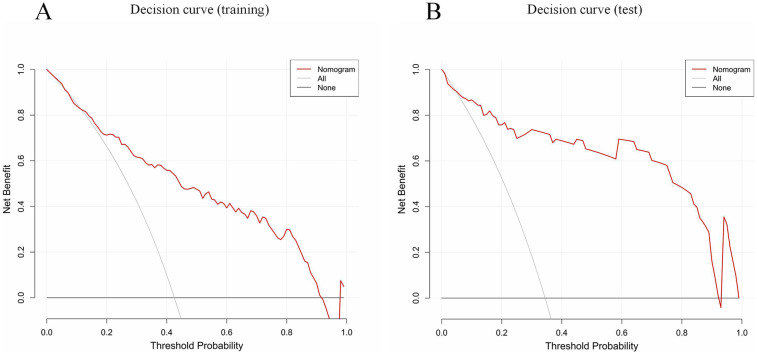
Decision curve analysis (DCA) of the CT-based nomogram (red line). All (gray solid line, this assumes you intervene on every single patient) and None (thick black line at *y* = 0, this assumes you do not intervene on any patient). The *x*-axis indicates the threshold probability. The *y*-axis indicates the net benefit. DCA comparing the net benefit of the nomogram in the training set **(A)** and test set **(B)**.

Calibration curves indicated good agreement between the predicted and actual outcomes (Figures 4C,D). The DCA of our nomogram shows that across a wide threshold probability range of approximately 0.1–0.9, its net benefit is consistently higher than the strategies of “intervening on all patients” and “intervening on no patients.” This indicates that in clinical practice, using this model to screen patients requiring early identification of MUMPP can provide greater net clinical benefit compared to simple binary strategies, effectively balancing the risks of missed diagnosis and overdiagnosis (Figure 5).

**Figure 6:**
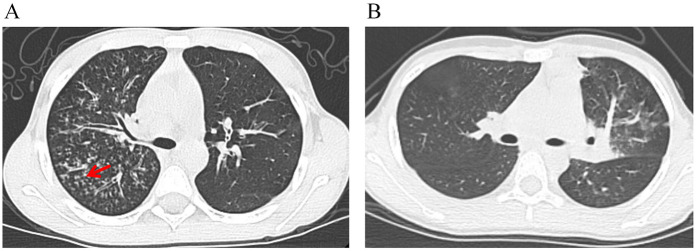
The risk of developing MUMPP was calculated for two pediatric patients using a nomogram. **(A)** A chest CT image from a boy with MPP shows the tree-in-bud sign (red arrow). His laboratory findings were as follows: NEUT-value, 10.22 × 10⁹/L; LYMPH-value, 1.98 × 10⁹/L; CK, 257 U/L; PLR, 267. The total nomogram score was approximately 158 points, corresponding to a probability of developing MUMPP of greater than 90%. **(B)** A chest CT image from a girl with MPP shows no tree-in-bud sign. Her laboratory findings were as follows: NEUT-value, 6.42 × 10⁹/L; LYMPH-value, 2.76 × 10⁹/L; CK, 197 U/L; PLR, 136. The total nomogram score was approximately 111 points, corresponding to a probability of developing MUMPP of less than 20%. NEUT-value, neutrophil count; LYMPH-value, lymphocyte count; CK, creatine kinase; PLR, platelet-to-lymphocyte ratio.

Figure 6 provides an example of how to use the nomogram. Similar to a scoring system, points are assigned for each predictor of MUMPP, which correspond to the risk of MUMPP. A vertical line can be drawn upward from each predictor to determine the points associated with the presence of the tree-in-bud sign on chest CT, gender, neutrophil count, lymphocyte count, creatine kinase, and platelet-to-lymphocyte ratio (PLR). Once points are assigned for all predictors, the total points are calculated. The total points are then converted into the probability of MUMPP by reading the corresponding value on the total points scale.

Consequently, we created an online computing platform (https://dynom.shinyapps.io/DynNomapp/). This platform enables both doctors and patients to carry out calculations directly on the web.

## Discussion

In recent years, the prevalence of MPP has increased significantly, with high rates of complications and mortality (15). Macrolides are the primary antibiotics used to treat MPP, however, due to the increased use of macrolides and the emergence of drug-resistant strains, the number of macrolide-resistant cases has been rising. This situation has become particularly complex following the COVID-19 pandemic, further complicating the clinical management of MUMPP in children (16, 17).

We found that a nomogram constructed using logistic regression, incorporating tree-in-bud pattern, lymphocyte-value, neutrophil-value, creatine kinase (CK), platelet-to-lymphocyte ratio (PLR), and sex, can effectively and conveniently predict MUMPP at an early stage. The nomogram demonstrated strong accuracy and discriminative ability, which indicated it may be a practical tool to help pediatricians recognize MUMPP earlier.

In our study, the tree-in-bud sign was identified as a significant independent predictor of MUMPP. The tree-in-bud sign, a CT pattern characterized by centrilobular nodules and branching linear opacities resembling a budding tree, reflects pathological changes in the small airways, including bronchiolar wall thickening, luminal impaction with mucus, pus, or granulation tissue, and peribronchiolar inflammation (18, 19). The pathogenesis of MUMPP involves not only direct microbial damage but also a robust host immune-inflammatory response, leading to a massive release of inflammatory cytokines and subsequent tissue injury (20, 21). A more pronounced immune response is associated with greater organ damage and dictates disease prognosis. In this context, a widespread tree-in-bud sign signifies extensive lung involvement and severe small airway obstruction (22, 23), which can impede pathogen clearance, reduce local antibiotic concentration, and ultimately diminish treatment responsiveness, as supported by previous findings (24). By incorporating this key imaging feature, our model facilitates the early recognition of MUMPP, thereby aiding in timely treatment adjustment, mitigating the risk of complications such as bronchiolitis obliterans, and improving long-term outcomes in children.

Peripheral blood markers provide important insights into the host's immune-inflammatory status. This study revealed significantly elevated neutrophil-value in children with MUMPP, identifying it as an independent risk factor. This aligns with previous studies suggesting that neutrophil-mediated hyperinflammatory responses are closely associated with MPP severity and poor outcomes (25–27). A possible explanation is that neutrophils, as the first line of defense against infection, are activated upon MP invasion via granulocyte colony-stimulating factor (G-CSF) produced in large quantities by bronchial epithelial cells, leading to a rapid increase in neutrophil counts and enhanced phagocytic activity (28). Persistent inflammation may result in lymphocyte-value depletion and impaired cellular immune function, which is supported by the decreased lymphocyte-value observed in the MUMPP group. Platelets are not only involved in coagulation but also act as important inflammatory effector cells. MP infection, along with associated cytokine storms and hypoxia, can cause endothelial injury, leading to platelet activation and consumption (29). Previous studies have shown that lower PLT levels are associated with a higher risk of SMPP (30). Although PLT alone did not differ significantly between groups in this study, a lower PLR was identified as a risk factor for MUMPP. As a composite indicator, PLR may more sensitively reflect concurrent platelet consumption and lymphopenia, indicating stronger inflammatory responses and potential immune imbalance, which correlate with disease severity (31).

Additionally, a higher proportion of male patients was observed in the MUMPP group. Although the relationship between sex and MUMPP remains underexplored, this phenomenon could potentially be due to variations in immune function or hormonal regulation between males and females (32). Elevated serum CK was also more common in children with MUMPP. CK is a key enzyme in energy metabolism within muscle cells, predominantly found in skeletal and cardiac muscle, with smaller amounts present in the brain, intestine, liver, spleen, and lungs. Under normal conditions, CK rarely leaks out of cells due to intact cell membranes. Higher CK levels in MUMPP may reflect extrapulmonary involvement (e.g., muscle damage) or a stronger systemic stress response (33).

Furthermore, The COVID-19 pandemic and its subsequent impacts have reshaped the epidemiological landscape and clinical management of MPP. Global studies indicate that public health interventions during the pandemic and shifts in population immunity created favorable conditions for the resurgence and spread of MPP, often accompanied by increased macrolide resistance (34). In terms of technological responses, breakthroughs in COVID-19 vaccine development—particularly highly stable and immunogenic circular RNA platforms—offer potential avenues for future strategies against drug-resistant *Mycoplasma* strains (35). Meanwhile, national surveillance data on fever of unknown origin (FUO) in China reveal that infectious diseases remain the leading cause of FUO in the post-pandemic era. However, the proportion of undiagnosed cases has risen, reflecting growing diagnostic complexity. Significant regional variations in etiology further underscore the need for individualized diagnosis tailored to local epidemiological patterns (36). Against this backdrop, our study addresses the diagnostic challenges of the post-COVID era by developing an objective prediction tool—the nomogram model presented here—for early identification of MUMPP. This tool aims to support more precise early clinical intervention in complex diagnostic scenarios.

Although the nomogram model established in this study has demonstrated favorable predictive performance, medical research is advancing toward an era driven by artificial intelligence (AI) (37). Future models may leverage deep learning to enable automated analysis of CT images and integrate multimodal data—including clinical features, laboratory indicators (such as the predictors identified in this study), genomic information, and radiomic features—to construct more powerful predictive models (38, 39). Such integrated models would not only predict MUMPP risk as achieved in the current study but also hold the potential for dynamic and individualized assessment of treatment response. By continuously monitoring changes in imaging and clinical data throughout the treatment process, these models could provide data-driven support for real-time adjustments to therapeutic strategies, thereby paving the way for personalized management of MPP.

There are also some limitations in this study. First, this study is retrospective in design, and all data were derived from a single medical center. Although an internal test set was used for validation, its relatively small sample size may lead to an overestimation of model performance. Consequently, the generalizability of our model may be limited when applied to other populations or healthcare institutions with different diagnostic and treatment standards. Second, regarding predictor selection, our model was developed primarily based on routine clinical and imaging indicators. It was thus developed without incorporating potentially influential microbiological factors, such as specific macrolide resistance-associated gene mutations, and does not account for the potential associations with a history of prior COVID-19 infection or vaccination status. The latter is particularly relevant as the post-pandemic immune landscape may influence the presentation and severity of other respiratory infections like MPP. Furthermore, the prediction model is constructed using traditional logistic regression methodology and has not yet incorporated more advanced AI modeling techniques, which might better capture complex, non-linear relationships. Finally, the clinical applicability and robustness of this nomogram require further validation through prospective, multi-center studies. The retrospective, single-center design may introduce selection bias, and the observed differences in baseline characteristics between the training and test cohorts highlight the need for validation in larger, more diverse populations to ensure generalizability.

## Conclusion

We developed and validated a simple-to-use nomogram for predicting MUMPP in early stage. The nomogram demonstrates strong discriminatory power and calibration, and may be a practical tool for clinical practice.

## Data Availability

The raw data supporting the conclusions of this article will be made available by the authors, without undue reservation.
