# Supplementary material for: Development and validation of a nomogram for early prediction of macrolide-unresponsive Mycoplasma pneumoniae pneumonia in children
Source: Front Pediatr. 2025 Nov 20;13:1695974. doi: 10.3389/fped.2025.1695974 (PMC12675469; doi:10.3389/fped.2025.1695974)
Supplement: Supplementary file 1 [file Datasheet1.pdf]

TRIPOD Checklist: Prediction Model Development and Validation

Section/Topic	Item		Checklist Item	Section headings
**Title and abstract**				
Title	1	D;V	Identify the study as developing and/or validating a multivariable prediction model, the target population, and the outcome to be predicted.	Title section
Abstract	2	D;V	Provide a summary of objectives, study design, setting, participants, sample size, predictors, outcome, statistical analysis, results, and conclusions.	Abstract section
**Introduction**				
Background and objectives	3a	D;V	Explain the medical context (including whether diagnostic or prognostic) and rationale for developing or validating the multivariable prediction model, including references to existing models.	Introduction section
	3b	D;V	Specify the objectives, including whether the study describes the development or validation of the model or both.	Introduction section
Methods				
Source of data	4a	D;V	Describe the study design or source of data (e.g., randomized trial, cohort, or registry data), separately for the development and validation data sets, if applicable.	Methods section, Study design and data source
	4b	D;V	Specify the key study dates, including start of accrual; end of accrual; and, if applicable, end of follow-up.	Methods section, Study design and data source
Participants	5a	D;V	Specify key elements of the study setting (e.g., primary care, secondary care, general population) including number and location of centres.	Methods section, Study design and data source
	5b	D;V	Describe eligibility criteria for participants.	Methods section, Inclusion and exclusion criteria
	5c	D;V	Give details of treatments received, if relevant.	Methods section, Outcome definition
Outcome	6a	D;V	Clearly define the outcome that is predicted by the prediction model, including how and when assessed.	Methods section, Outcome definition
	6b	D;V	Report any actions to blind assessment of the outcome to be predicted.	Not Applicable
Predictors	7a	D;V	Clearly define all predictors used in developing or validating the multivariable prediction model, including how and when they were measured.	Methods section, Data collection and Imaging analyses
	7b	D;V	Report any actions to blind assessment of predictors for the outcome and other predictors.	Not applicable
Sample size	8	D;V	Explain how the study size was arrived at.	Methods section, Study design and data source
Missing data	9	D;V	Describe how missing data were handled (e.g., complete-case analysis, single imputation, multiple imputation) with details of any imputation method.	Methods section, Study design and data source
Statistical analysis methods	10a	D	Describe how predictors were handled in the analyses.	Methods section, Statistical analysis
	10b	D	Specify type of model, all model-building procedures (including any predictor selection), and method for internal validation.	Methods section, Statistical analysis
	10c	V	For validation, describe how the predictions were calculated.	Methods section, Statistical analysis
	10d	D;V	Specify all measures used to assess model performance and, if relevant, to compare multiple models.	Methods section, Statistical analysis
	10e	V	Describe any model updating (e.g., recalibration) arising from the validation, if done.	Not applicable
Risk groups	11	D;V	Provide details on how risk groups were created, if done.	Not applicable
Development vs. validation	12	V	For validation, identify any differences from the development data in setting, eligibility criteria, outcome, and predictors.	Methods section, Study design and data source
Results				
Participants	13a	D;V	Describe the flow of participants through the study, including the number of participants with and without the outcome and, if applicable, a summary of the follow-up time. A diagram may be helpful.	Results section, Clinical characteristics
	13b	D;V	Describe the characteristics of the participants (basic demographics, clinical features, available predictors), including the number of participants with missing data for predictors and outcome.	Results section, Clinical characteristics
	13c	V	For validation, show a comparison with the development data of the distribution of important variables (demographics, predictors and outcome).	Results section, Clinical characteristics
Model development	14a	D	Specify the number of participants and outcome events in each analysis.	Results section, Clinical characteristics
	14b	D	If done, report the unadjusted association between each candidate predictor and outcome.	Results section, Variable selection
Model specification	15a	D	Present the full prediction model to allow predictions for individuals (i.e., all regression coefficients, and model intercept or baseline survival at a given time point).	Results section, Development and Evaluation of the Predictive Nomogram
	15b	D	Explain how to the use the prediction model.	Results section, Development and Evaluation of the Predictive Nomogram

Model performance	16	D;V	Report performance measures (with CIs) for the prediction model.	Results section, Development and Evaluation of the Predictive Nomogram
Model-updating	17	V	If done, report the results from any model updating (i.e., model specification, model performance).	Not applicable
**Discussion**				
Limitations	18	D;V	Discuss any limitations of the study (such as nonrepresentative sample, few events per predictor, missing data).	Discussion section, Limitations
Interpretation	19a	V	For validation, discuss the results with reference to performance in the development data, and any other validation data.	Discussion section, Interpretation
	19b	D;V	Give an overall interpretation of the results, considering objectives, limitations, results from similar studies, and other relevant evidence.	Discussion section, Interpretation
Implications	20	D;V	Discuss the potential clinical use of the model and implications for future research.	Discussion section, Implications
Other information				
Supplementary information	21	D;V	Provide information about the availability of supplementary resources, such as study protocol, Web calculator, and data sets.	Discussion section, Online platform
Funding	22	D;V	Give the source of funding and the role of the funders for the present study.	Acknowledgments

*Items relevant only to the development of a prediction model are denoted by D, items relating solely to a validation of a prediction model are denoted by V, and items relating to both are denoted D;V. We recommend using the TRIPOD Checklist in conjunction with the TRIPOD Explanation and Elaboration document.
