# Supplementary material for: Development and validation of a nomogram for early prediction of macrolide-unresponsive Mycoplasma pneumoniae pneumonia in children
Source: Front Pediatr. 2025 Nov 20;13:1695974. doi: 10.3389/fped.2025.1695974 (PMC12675469; doi:10.3389/fped.2025.1695974)
Supplement: Supplementary file 2 [file Table1.docx]

# **Table S1**

| Variable | Mean | Median | SD | Skewness |
| --- | --- | --- | --- | --- |
| NEUT_value | 4.823 | 4.375 | 2.369 | 1.939 |
| LYMPH_value | 1.935 | 1.685 | 1.22 | 3.146 |
| CK | 130.287 | 90 | 174.702 | 6.378 |
| PLR | 176.629 | 156.625 | 94.651 | 1.481 |
