# Supplementary material for: Development and validation of a nomogram for early prediction of macrolide-unresponsive Mycoplasma pneumoniae pneumonia in children
Source: Front Pediatr. 2025 Nov 20;13:1695974. doi: 10.3389/fped.2025.1695974 (PMC12675469; doi:10.3389/fped.2025.1695974)
Supplement: Supplementary file 3 [file Table2.docx]

# **Table S2**

| Variable | Factor Chi-Square | Factor d.f. | Factor P-value | Nonlinear Chi-Square | Nonlinear d.f. | Nonlinear P-value | Total Chi-Square | Total d.f. | Total P-value | Linearity Conclusion |
| --- | --- | --- | --- | --- | --- | --- | --- | --- | --- | --- |
| NEUT_value | 7.99 | 3 | 0.0463 | 3.99 | 2 | 0.1361 | 7.99 | 3 | 0.0463 | Linear |
| LYMPH_value | 19.49 | 3 | 0.0002 | 0.56 | 2 | 0.7574 | 19.49 | 3 | 0.0002 | Linear |
| CK | 13 | 3 | 0.0046 | 0.4 | 2 | 0.8178 | 13 | 3 | 0.0046 | Linear |
| PLR | 11.69 | 3 | 0.0085 | 2.35 | 2 | 0.3089 | 11.69 | 3 | 0.0085 | Linear |
