# Supplementary material for: Development and validation of a nomogram for early prediction of macrolide-unresponsive Mycoplasma pneumoniae pneumonia in children
Source: Front Pediatr. 2025 Nov 20;13:1695974. doi: 10.3389/fped.2025.1695974 (PMC12675469; doi:10.3389/fped.2025.1695974)
Supplement: Supplementary file 4 [file Table3.docx]

**Table** S3 The results of validation

| Metric | Original | Optimism | Optimism-corrected |
| --- | --- | --- | --- |
| Dxy (discrimination) | 0.676 | 0.042 | 0.634 |
| R² (explained variation) | 0.436 | 0.054 | 0.382 |
| Calibration slope | 1.000 | 0.121 | 0.879 |
| Calibration intercept | 0.000 | 0.021 | -0.021 |

Note that the Dxy index is directly related to the C-index via the formula: C-index = (Dxy + 1)/2. Based on this, the optimism-corrected C-index was calculated as 0.817.

The optimism-corrected metrics indicate that, after accounting for potential overfitting, the model maintains good discriminative ability (as reflected by the C-index of 0.817) and acceptable calibration (evidenced by the calibration slope of 0.879 and intercept of -0.021).

**Results：**

Internal validation via bootstrap with 1000 resamples yielded the following optimism-corrected performance metrics:

1，Dxy = 0.634 (corresponding to a C-index of 0.817), indicating good discriminative ability;

2.R² = 0.382, reflecting the extent of explained variation;

3.Calibration slope = 0.879 and calibration intercept = -0.021, indicating acceptable calibration.

These findings confirm that the model maintains robust performance after correcting for potential overfitting.
